# Polyphenol-Rich and Alcoholic Beverages and Metabolic Status in Adults Living in Sicily, Southern Italy

**DOI:** 10.3390/foods10020383

**Published:** 2021-02-09

**Authors:** Agnieszka Micek, Justyna Godos, Achille Cernigliaro, Raffaele Ivan Cincione, Silvio Buscemi, Massimo Libra, Fabio Galvano, Giuseppe Grosso

**Affiliations:** 1 Department of Nursing Management and Epidemiology Nursing, Faculty of Health Sciences, Jagiellonian University Medical College, 31-501 Krakow, Poland; 2 Department of Biomedical and Biotechnological Sciences, University of Catania, 95123 Catania, Italy; justyna.godos@gmail.com (J.G.); m.libra@unict.it (M.L.); fgalvano@unict.it (F.G.); giuseppe.grosso@unict.it (G.G.); 3 Department of Health Service and Epidemiological Observatory, Health Authority Sicily Region, 90145 Palermo, Italy; achille.cernigliaro@regione.sicilia.it; 4 Department of Clinical and Experimental Medicine, University of Foggia, 71122 Foggia, Italy; ivan.cincione@unifg.it; 5 Biomedical Department of Internal and Specialist Medicine (DIBIMIS), University of Palermo, 90123 Palermo, Italy; silvio.buscemi@unipa.it; 6 Research Center for Prevention, Diagnosis and Treatment of Cancer, University of Catania, 95123 Catania, Italy

**Keywords:** beverages, polyphenols, phenolic acids, flavonoids, hydroxycinnamic acids, chlorogenic acid, wine, beer, coffee, tea, juice

## Abstract

Polyphenol-rich beverage consumption is not univocally accepted as a risk modulator for cardio-metabolic risk factors, despite mechanistic and epidemiological evidence suggesting otherwise. The aim of this study was to assess whether an association between polyphenol-rich beverage consumption and metabolic status could be observed in a Mediterranean cohort with relatively low intake of tea, coffee, red and white wine, beer, and fresh citrus juice. Demographic and dietary characteristics of 2044 adults living in southern Italy were analyzed. Multivariate logistic regression analyses were used to calculate odds ratios (ORs) and 95% confidence intervals (CIs) of the association between polyphenol-rich and alcoholic beverage consumption and metabolic status adjusted for potential confounding factors. Specific polyphenol-rich beverages were associated, to a various extent, with metabolic outcomes. Individuals with a higher total polyphenol-rich beverages had higher polyphenols intake and were less likely to have hypertension, type-2 diabetes, and dyslipidemia (OR = 0.57, 95% CI: 0.44–0.73; OR = 0.41, 95% CI: 0.26–0.66; and OR = 0.41, 95% CI: 0.29–0.57, respectively). However, when adjusted for potential confounding factors, only the association with hypertension remained significant (OR = 0.69, 95% CI: 0.50–0.94). Current scientific evidence suggests that such beverages may play a role on cardio-metabolic risk factors, especially when consumed within the context of a dietary pattern characterized by an intake of a plurality of them. However, these associations might be mediated by an overall healthier lifestyle.

## 1. Introduction

Over the last 10 years, dietary factors have been shown to play a major role in affecting the risk of non-communicable diseases, accounting for 3.5 million deaths in 2019 according to the last report of the Global Burden of Disease study group [1]. Dietary risks which have been calculated as having the burden of disease have strong scientific evidence and quantifiable risk measures associated with selected conditions, including cardiovascular diseases and cancers [1]. Such risks include a diet poor in plant-based foods (i.e., fruit and vegetables), whole grains and fibers, milk and calcium, and excess meat (red and processed) and sodium [2]; all these factors are supposed to contribute, to various extents, to the global burden of diseases [2]. However, while in line with general guidelines and international recommendations, other dietary factors of potential interests need to be further investigated to be correctly integrated in such scenarios. Specifically, aside from their content in fibers, vitamins and essential fatty acids, certain plant-based foods are known to be rich in antioxidant phytochemicals, such as polyphenol compounds [3]. Polyphenols have been associated with reduced risk of mortality [4], with stronger evidence on cardiovascular disease [5] rather than cancer risk [6]. Whether or not these compounds might exert beneficial effects on humans, it will be important to integrate current evidence taking into account beverage-rich polyphenols generally derived from fruits, seeds, or herbs.

There is a growing evidence for the health benefits of polyphenol-rich beverages [7]. Red wine has been long investigated to potentially exert cardiovascular benefits through a number of mechanisms, including, among others, improvement of endothelial function [8]. At least part of these potential effects have been attributed to its content in resveratrol, a polyphenol compound belonging to the stilbenes group, which has been shown to have anti-diabetic, anti-hypertensive, and blood lipid-lowering properties, especially in laboratory studies [9,10], while other evidence has been drawn in a human clinical setting [11,12,13]. Furthermore, several studies have demonstrated the favorable effects of white wine intake toward cardiovascular health through modulation of oxidative stress and metabolic biomarkers [14]. However, similar associations with decreased risk of cardiovascular disease have been reported for other alcoholic beverages as well, including beer and spirits, opening the question as to whether other polyphenol groups might have the same effects, or if alcohol content might be responsible for the aforementioned observational findings. Tea and, more recently, coffee have also demonstrated potential benefits towards several aspects related to cardio-metabolic and endothelial health [15,16]. The highest represented polyphenol groups associated with consumption of such beverages are flavonoids (specifically catechins belonging to the group of flavanols) and phenolic acids (specifically caffeic, ferulic, and chlorogenic acids belonging to the group of hydroxycinnamic acids) [17]. Compared to other dietary compounds, there is an enormous variability in polyphenol consumption across European populations, both in terms of quantity and quality [18]. There is evidence from epidemiological studies that in Eastern Europe such variability is strongly influenced by polyphenol-rich beverage consumption rather than other food groups (i.e., fruits), as the content in polyphenols is more sensitive to variation in the former rather than the latter [19]. Concerning the Southern Italian population, it has been reported that consumption of polyphenol-rich beverages is relatively low [20]: The main reasons for this rely on the lack of such beverages in traditional dietary patterns (i.e., tea), different preparations (i.e., consumption of short espresso instead of long coffees), or patterns of consumption (i.e., daily moderate wine during meals vs. binge drinking over weekends) [21]. Thus, to investigate whether moderate-to-low consumption of polyphenol-rich and alcoholic beverages may be associated with metabolic outcomes, the aim of this study was to test this potential association in a cohort of Southern Italian adults.

## 2. Materials and Methods

### 2.1. Study Population

The Mediterranean healthy Eating, Aging and Lifestyle (MEAL) study is an observational study conducted in Mediterranean island of Sicily aimed to investigate the association between traditional nutritional and lifestyle habits and non-communicable diseases. A detailed description of the study protocol was posted elsewhere [22]. In brief, between 2014 and 2015 the inhabitants of the main districts of the Catania, the city located in the southern region of Italy, were chosen at random to participate in a study. The enrolment and data collection was conducted in age- (10-year) and sex-specific strata groups based on the registered records of local general practitioners. An a priori calculated sample size of 1500 individuals was planned to assure a relative precision of 5% (Type I and II errors of 0.05 and 0.10, respectively), anticipating a participation rate of 70%. Out of 2405 individuals invited, the final sample comprised 2044 participants (response rate of 85%). All participants were informed about the aims of the study and provided a written informed consent. All the study procedures were carried out in accordance with the Declaration of Helsinki (1989) of the World Medical Association. The study protocol has been reviewed and approved by the concerning ethical committee.

### 2.2. Data Collection

During the face-to-face personal interviews, with the use of tablet computers, an electronic data collection was implemented. All the participants were administered with a paper copy of the questionnaire to better visualize the response options, but yet the interviewer noted down the final answers personally. The following background data were collected: sex, age at recruitment, highest degree of education, physical activity level, and smoking status. Educational status was categorized as (i) low (primary/secondary), (ii) medium (high school), and (iii) high (university). International Physical Activity Questionnaires (IPAQ) [23] allowed to estimate last 7-day physical activity in 5 domains and based on its final score, physical activity was grouped at (i) low, (ii) moderate, and (iii) high. Smoking status was classified as being a never, ex- or current smoker, while alcohol consumption was categorized as (i) none, (ii) moderate (0.1–12 g/d) and (iii) regular (>12 g/d) drinker. Anthropometric assessments were undertaken according to the standardized techniques [24]. Body height was measured with precision of 0.5 cm in barefoot participants with a right-angle triangle resting on the scalp, who were standing back to wall and focusing eyes straight ahead. Body mass index (BMI) was categorized as under or normal weight (BMI <25 kg/m^2^), overweight (BMI 25 to 29.9 kg/m^2^) and obese (BMI ≥ 30 kg/m^2^).

### 2.3. Dietary Assessment

In order to investigate the dietary intake, two versions of food frequency questionnaires (FFQs), a long and a short, were administered [25,26]. The validity and reliability of both of them have been previously tested in the Sicilian population. Intake of seasonal foods referred to consumption during the period in which the food was available and then adjusted by its proportional intake in one year. Food composition tables of the Italian Research Center for Foods and Nutrition (accessed on 17 July 2020 and available online at https://www.crea.gov.it/-/tabella-di-composizione-degli-alimenti) were applied to estimate the total energy content and a specific food intake as well as to calculate the micro- and macro-nutrients content (based on the comparison of both databases). The process of the estimation of habitual polyphenol intake has been retrieved from the Phenol-Explorer database (accessed on 17 July 2020 and available online at www.phenol-explorer.eu) as previously described in detail [27]. The individual food consumption (in ml or g) was obtained for each participant of the study by following the standard portion sizes and then was extrapolated to 24 h intake; next, the databases were searched to retrieve mean content values for macro-, micronutrients and polyphenols contained in the foods obtained and the nutrient and polyphenol intake from each food was calculated by multiplying the content by the daily consumption of each food adjusted for total energy intake (kcal/d) using the residual method [21]. FFQs with lacking data or unreliable intakes (<1000 or >6000 kcal/d) were excluded from the analyses (*n* = 198) leaving a total of 1846 individuals included in the analysis. The variables of interest were intake of tea, coffee (espresso/stove-top), red and white wine, beer, and fruit juice (fresh citrus juice).

Adherence to the Mediterranean diet was assessed using a literature-based score not including nut consumption among its criteria [28]. The score was built assigning positive points for consumption of food groups supposed to represent adherence to the Mediterranean diet, such as fruit, vegetables, legumes, cereals, fish, and olive oil, and negative points for excess consumption of food groups not representing it, such as meat and dairy products; moderate alcohol intake was deemed as optimal for higher adherence. The final adherence score comprises nine food categories with a score ranging from 0 point (lowest adherence) to 18 points (highest adherence) and were individuals grouped in tertiles and categorized as low, medium, and high adherents of the Mediterranean diet.

### 2.4. Metabolic Outcomes

Arterial blood pressure was measured three times at the right arm relaxed and well supported by a table, with an angle of 45 degree from the trunk at the end of the physical examination with the subject in sitting position and at least 5 min at rest. A mean of the last two measurements was calculated and considered for inclusion in the database. Information from measurements was integrated with general practitioners computerized records, as specialist diagnoses were required for patients with disease in order that they could obtain drugs. Patients have been considered hypertensive when average systolic/diastolic blood pressure levels were greater or equal to 140/90 mm Hg, medical history of taking anti-hypertensive medications, or based on the previous diagnosis of hypertension. Patients were considered diabetic or dyslipidemic whether previously diagnosed with diabetes and hypercholesterolemia/hypertriglyceridemia, respectively.

### 2.5. Statistical Analysis

Absolute numbers and percentages are used to describe categorical variables, means and standard deviations are used to describe continuous variables. Individuals were grouped by tertile of polyphenol-rich beverages for each exposure and distribution of background characteristics and polyphenol intake were compared between groups. Differences were tested with Chi-squared test for categorical variables, ANOVA for continuous variables distributed normally, and Kruskall–Wallis test for variables distributed not normally. Energy-adjusted multivariate logistic regression models were used to calculate odds ratios (ORs) and 95% confidence intervals (CIs) for association between tertiles of individual polyphenol-rich beverage intakes and metabolic outcomes. A multivariate model adjusted for all other background characteristics (BMI, physical activity, educational status, smoking status) was also performed in order to test whether the observed associations were independent from the aforementioned variables. An additional model further adjusting for adherence to the Mediterranean diet was used to test whether the retrieved associations were independent of the overall quality of the diet.

Since a simple sum of quantity of beverage intake could not be performed, based on the initial grouping by tertile for each beverage, a sample-level grouping by polyphenol-rich beverage consumption was done by ranking individuals as “low intake” if they scored in the first tertile of intake for all beverages, “medium intake” if they scored in at least 2 beverages in the second tertile, and “high intake” if they scored in third tertile in at least 2 beverages. This grouping was arbitrary, but allowed a roughly equal distribution of the sample across the 3 categories (n = 441, 806, and 600 individuals in low, medium, and high intake groups, respectively) taking into account the quantity of intake irrespectively of the type of beverage. In order to test whether the total polyphenol-rich beverage consumption was related to other dietary variables, including polyphenol intake, a comparison of mean macro- and micronutrients, and polyphenols by the 3 groups of total polyphenol-rich beverage intakes was also conducted. Uni- and multivariate regression models as previously described were finally performed to test the association between total polyphenol-rich beverage consumption and metabolic outcomes.

All reported P-values were based on two-sided tests, and significance level was 5%. SPSS 21 (SPSS Inc., Chicago, IL, USA) software was used for all the statistical analysis.

## 3. Results

The mean intake of individual polyphenol-rich beverages in each tertile group is reported in Supplementary Table S1: the mean intake in the first tertile was nearly no consumption; in the second tertile, the mean intake was also relatively low consumption of such beverages (roughly one drink per week) with the exception of coffee (about 1 cup per day); the mean intake in the third tertile varied depending on the beverage, accounting for about one drink per day of tea, beer, fresh citrus juice, 2 drinks per day of red wine, 3 cups of coffee per day, and about 1 drink per week of white wine.

The distribution of background characteristics by tertiles of polyphenol-rich and alcoholic (alcoholic beverages containing polyphenols) beverages is shown in Table 1 and Table 2, respectively. There were no clear trends or patterns in distribution of background variables for all polyphenol-rich beverages. There was a higher proportion of women among the highest and lowest consumers of tea; older individuals seemed to be more frequent among red wine consumers while younger among tea and fresh citrus juice consumers; higher educated individuals among wine, beer, and fresh citrus juice consumers but also in low coffee and red wine consumers; a higher proportion of current and former smokers was found among higher coffee, wine, and beer drinkers, while there was a higher proportion of non-smokers among higher consumers of fresh citrus juices; regarding physical activity levels, a non-linear distribution across categories was observed for tea and coffee (high consumers among low and high physical activity level) and a linear increasing levels with higher intake of wine (red and white), beer and fresh citrus juice; finally, there was a lower proportion of obese individuals among higher consumers of all beverages with the exception of coffee (Table 1 and Table 2). Concerning adherence to the Mediterranean diet, a higher proportion of high adherent individuals was found among higher consumers of wine (red and white), beer, and fresh citrus juice (Table 1 and Table 2).

Intake of total and major groups of polyphenols by intake of individual polyphenol-rich and alcoholic (alcoholic beverages containing polyphenols) beverages is shown in Table 3 and Table 4. While higher consumption of polyphenol-rich beverages reflected higher total polyphenol intake with the exception for coffee and fresh citrus juice, no univocal patterns or trends could have been observed concerning individual polyphenol groups, as each type of beverage has a specific content type of polyphenols: higher tea consumers had higher intake of flavonoids and phenolic acids; higher coffee consumers had a lower intake of flavonoids and higher consumption of phenolic acids and stilbenes; higher red and white wine consumers had a higher intake of all polyphenol groups; higher beer consumers had higher phenolic acids; and higher fresh citrus juice consumers had lower stilbenes (Table 3 and Table 4). 

Table 5 and Table 6 show the association between individual type of polyphenol-rich and alcoholic (alcoholic beverages containing polyphenols) beverage intake and metabolic outcomes. After adjustment for covariates and adherence to Mediterranean diet, higher coffee intake was inversely associated with hypertension and dyslipidemia (OR = 0.64, 95% CI: 0.48–0.86 and OR = 0.64, 95% CI: 0.43–0.95, respectively); red wine was inversely associated with all outcomes, despite concerning type-2 diabetes and dyslipidemia the significant association was reached only in the medium group of intake (OR of hypertension for high vs. low intake = 0.61, 95% CI: 0.41–0.90; OR of type-2 diabetes for medium vs. low intake = 0.37, 95% CI: 0.19–0.71; OR of dyslipidemia for medium vs. low intake = 0.39, 95% CI: 0.24–0.63); higher beer intake was inversely associated with hypertension and type-2 diabetes (OR = 0.61, 95% CI: 0.45–0.83 and OR = 0.51, 95% CI: 0.28–0.92, respectively); finally, higher fresh citrus juice intake was inversely associated with type-2 diabetes and dyslipidemia (OR = 0.43, 95% CI: 0.23–0.80 and OR = 0.32, 95% CI: 0.22–0.48, respectively).

The sample ranking by total consumption of polyphenol-rich beverages (irrespective of the type) showed a good distribution across the 3 groups of all beverages except for tea, which eventually was consumed significantly only by a small number of individuals that did not consume as many other beverages (Table 7). Among macronutrients, no trends were observed for carbohydrates and proteins, while fats increased with the increasing intake of beverages (Table 7). Moreover, intake of some micronutrients and minerals followed the trend of polyphenol-rich beverage consumption, vitamin C, vitamin E, sodium, and potassium (Table 7). Conversely, intake of total and all major groups of polyphenols except for lignans were higher with increasing intake of polyphenol-rich beverages (Table 7).

Table 8 finally shows the association between ranking of polyphenol-rich beverage consumption and metabolic outcomes. In the energy-adjusted model, the highest intake of polyphenol-rich beverages was associated with all metabolic outcomes investigated (OR = 0.57, 95% CI: 0.44–0.73; OR = 0.41, 95% CI: 0.26–0.66; and OR = 0.41, 95% CI: 0.29–0.57 for hypertension, type-2 diabetes, and dyslipidemia, respectively). However, when adjusted for background characteristics, only the association with hypertension remained significant (Table 5); the retrieved odds did not change when further adjusting for adherence to the Mediterranean diet, remaining significant only for hypertension (OR = 0.69, 95% CI: 0.50–0.94).

## 4. Discussion

This study aimed to assess whether an association between polyphenol-rich beverages and metabolic outcomes could be observed in a southern Italian population. The results obtained showed that all polyphenol-rich beverages were associated, to a varying extent, with metabolic outcomes. In the attempt to explore the role of all beverages, individuals were ranked based on the tertile of consumption irrespective of their individual beverage. Interestingly, while individual types of polyphenol-rich beverages led to a higher intake of specific polyphenol groups in line with their individual content, this was not significant for all beverages; when ranking the sample for total intake of polyphenol-rich beverages, the groups scoring with the highest consumption had a higher intake of all polyphenol groups. However, despite the association of total consumption of such beverages, this was significant in the least adjusted statistical model and adjustment for background characteristics led to a significant inverse association only with the occurrence of hypertension. We did not observe a potential healthier diet followed by individuals with higher intake of polyphenol-rich beverages, and the adjustment for adherence to the Mediterranean diet did not change the previous retrieved odds ratios; thus, we can only hypothesize that other background factors may play a role in the association between polyphenol-rich beverages and metabolic outcomes. These results remark some studies conducted in Mediterranean countries showing better markers of inflammation, lower occurrence of metabolic syndrome or cardiovascular diseases for higher consumption of espresso coffee [29,30,31] and red wine [32,33]. Several other studies showed the detrimental effects of beer and alcohol at higher doses, but no specific outcomes with low to moderate intake was demonstrated [34,35]. Despite our results being in line with the scientific literature providing explanation of selected mechanisms, some considerations are worth discussing in order to better interpret significant and null results and to design better future studies on this topic.

Consumption of both coffee and tea is relatively low in the Mediterranean population compared to north-eastern European countries [36]; despite this, there are no documented reasons for this besides cultural heritage, and it is possible that the differences in climate may play a role in such dietary choices regarding tea, while preference of espresso over regular coffee (rather not consumed at all in Italy) may justify the lower intake of coffee. Nonetheless, while no findings were reported for tea, individuals consuming a higher amount of coffee were less likely to have hypertension and dyslipidemia. Both conditions have been documented to show different associations with coffee consumption in an acute or chronic background: in fact, while clinical trials showed an increase in blood lipids and blood pressure [37], meta-analyses of prospective studies showed a decrease in risk of hypertension for higher intake of coffee in a dose-response manner [38], while no relation has been reported for dyslipidemia risk [39]; it is worth noting that both associations have been demonstrated to be modified by individuals’ genetic variants [39,40]. Mechanisms in support of such findings are mostly ascribable to the coffee content in phenolic acids and, more specifically, hydroxybenzoic acids (caffeic acid, ferulic acid and p-coumaric acid): their potential as anti-inflammatory and anti-hypertensive agents has been reported [41,42] while their metabolites attenuate oxidative stress (reactive oxygen species), which ultimately leads to the benefit of blood-pressure reduction through improved endothelial function and nitric oxide bioavailability in the arterial vasculature [43]. Among hydroxybenzoic acids, major attention to chlorogenic acid has been paid because provided most entirely only by coffee consumption and has been consistently reported to exert antioxidant activity toward vascular endothelial cells, in which the endothelial nitric oxide synthase expression may be promoted [44,45]. It has also been postulated that phenolic acids contained in coffee might be able to exert pivotal roles on lipid metabolism regulation [46]. Specific mechanisms may involve the interaction with intestinal microbe diversity, which in turn is associated with immune system function, inflammation and ultimately to metabolic health [47,48]. Regarding the null results on diabetes, we may hypothesize that doses of coffee might not be sufficient to reduce the risk as reported in the scientific literature, or unmeasured confounding factors (i.e., the use of sugar in coffee) might counteract its beneficial effects on insulin sensitivity and glucose homeostasis. Regarding tea, summary of evidence showed it does not appear to significantly affect blood pressure or lipids in short-term [49], while data on metabolic outcomes are mixed and seems to potentially depend on type of tea and doses (i.e., associations observed for higher intake than those reported in our study, which may explain lack of significant findings) [16,50].

Concerning alcoholic beverages, aside from spirits not explored in this study, there is an active debate on whether they should be considered harmful at any doses or exert potential preventive action against cardiovascular disease when consumed moderately [51,52]. Albeit a recent study of the Global Burden of Disease reported the impact of alcohol consumption (measured as harmonized exposure to one standard drink) on disability-adjusted life years and number of deaths, it fails discriminating type of alcohol, frequency (i.e., once per day vs. seven drinks in a day over a week), and occasion (i.e., during meals vs. overnight) [53]. There is a long tradition supporting the moderate consumption of alcohol (especially red wine) in the context of healthy dietary patterns, such as the Mediterranean diet [14], while scientific evidence from meta-analyses shows a clear J-curve association with risk of cardiovascular disease and mortality [54,55,56]. Alcohol consumption is strongly affected by the cultural heritage of a population, thus it is not surprising, although it is partially in contrast with the literature, that in the present study the intake of red wine, beer, and white wine (to a lesser extent), have been found to be associated with lower odds of some of the metabolic outcomes investigated. Notably, it is important to underline the relatively low doses for which we observed such results, meaning roughly one glass of red wine or beer per day or one glass of white wine per week; since we refer to estimation from FFQs, it is also likely that on a daily basis an individual would consume only one of the aforementioned beverages. Based on previous studies on this topic, it is possible that the “low dose” would be, in fact, a surrogate variable for the occasion of drinking (moderate, sporadic during meals) as compared to heavy binge drinking over weekends [57,58,59]. Although still the center of an actual debate, there is convincing evidence that moderate alcoholic beverages consumed during meals may exert positive effects on cardiovascular health [60]. All such alcoholic beverages contain different polyphenols that have been reported, to a various extent, to play a role in LDL oxidation, platelet aggregation, endothelial function and smooth muscle cell proliferation exerting antioxidant, anti-inflammatory, antithrombotic, and antiatherogenic actions [61]; moreover, low doses of alcohol per se may also rise blood HDL-cholesterol levels [61]. Thus, it is advisable that future studies start taking into consideration potential differences across countries relative to patterns of alcohol consumption, emphasizing the potential role of low doses of alcohol intake against cardio-metabolic risk factors.

In the present study we also investigated the potential association between fresh citrus juice consumption and cardio-metabolic risk factors, reporting lower odds of type-2 diabetes and dyslipidemia associated with their higher consumption. Citrus fruits are naturally rich in flavonoids, which have been reported to highly contribute to the total amount of polyphenols consumed in Southern European populations [18]. Among the main citrus flavonoids, hesperidin and naringin have consistently demonstrated the ability to modulate cell signal cascades as well as to influence the microbiota composition and activity, and exert beneficial effects toward intestinal barrier function. Antioxidant and anti-inflammatory effects [62,63] exerted by citrus flavonoids have been implicated in the prevention of type-2 diabetes and dyslipidemia [64,65]. Consumption of citrus fruit juices have been reported to potentially be an efficient way to improve consumption of fruits while reducing the risk of cardiovascular diseases mediated by antioxidant and anti-inflammatory effects, inhibition of platelet aggregation and improving vascular health [66,67]. Although concerns on sugar content and lack of fiber have been raised, the content in polyphenols may still be an advantage compared to other sorts of beverages. Thus, integration of fruit intake with fruit juice, especially citrus juice, could be a strategy to increase the amount of flavonoid intake and potentially exert benefits toward cardio-metabolic health.

The results of the present study should be considered in light of some important limitations. This was a cross-sectional study; thus, the cause–effect relation cannot be concluded. However, the aim of the study is to report data on the relation between polyphenol-rich beverages and corroborate their potential contribution when describing dietary risk factors from chronic non-communicable diseases. Another limitation is related to the assessment of dietary exposure: despite no golden standard existing and each technique to retrieve dietary information being affected by some limitations (i.e., recall bias, over or underestimation, etc.), the use of FFQs is widely adopted in cohort studies and is considered a reliable source of information for such investigations. Finally, the distribution of some variables was wide (with large SDs), and thus larger cohorts are needed to confirm the results retrieved.

## 5. Conclusions

In conclusion, polyphenol-rich beverages have been long investigated for potential health implications. Current scientific evidence suggests that such beverages may play a role in cardio-metabolic risk factors. In this study, individuals consuming more polyphenol-rich beverages were less likely to have hypertension, and to a various extent also type-2 diabetes and dyslipidemia. Consumption of all other polyphenol-rich beverages investigated, except coffee, was related to higher education, weight status and a more health-conscious profile should be taken into account when considering the results; in contrast, coffee consumption was associated with better health outcomes irrespective of the unfavorable background characteristics. Concerning alcoholic beverages, even though the results retrieved referred to relatively low consumption, diets characterized by consumption of polyphenol-rich beverages were in general high in polyphenols. Therefore, further and more accurate investigations are needed when studying the role of alcoholic beverages containing polyphenols on cardio-metabolic risk factors, taking into account that nationality of population may play a role in the pattern of consumption and ultimately its relationship with health.

## Figures and Tables

**Table 1:** Background characteristics of the study sample by consumption of individual polyphenol-rich beverages.

	Tea		Coffee		Fresh Citrus Juice	
	T1	T3	T1	T3	T1	T3
**Sex**						
Male	283 (44.6)	233 (34.1)	229 (45.1)	321 (40.6)	271 (39.6)	286 (42.6)
Female	351 (55.4)	451 (65.9) **	279 (54.9)	470 (59.4)	414 (60.4)	385 (57.4)
**Age group**						
<30	104 (16.4)	134 (19.6)	122 (24.0)	131 (16.6)	109 (15.9)	138 (20.6)
30–44	185 (29.2)	182 (26.6)	154 (30.3)	203 (25.7)	150 (21.9)	199 (29.7)
44–65	216 (34.1)	265 (38.7)	154 (30.3)	318 (40.2)	245 (35.7)	249 (37.1)
>65	129 (20.3)	104 (15.2) *	78 (15.4)	139 (17.6) **	182 (26.5)	85 (12.7) **
**Educational level**						
Low	239 (37.7)	239 (34.9)	151 (29.7)	309 (39.1)	337 (49.2)	178 (26.5)
Medium	252 (39.7)	246 (36.0)	214 (42.1)	289 (36.5)	203 (29.6)	290 (43.2)
High	143 (22.6)	199 (29.1)	143 (28.1)	193 (24.4) *	145 (21.2)	203 (30.3) **
**Smoking status**						
Non-smoker	378 (59.6)	431 (63.0)	369 (72.6)	427 (54.0)	383 (55.9)	442 (65.9)
Current smoker	154 (24.3)	170 (24.9)	83 (16.3)	224 (28.3)	160 (23.4)	153 (22.8)
Former smoker	102 (16.1)	83 (12.1)	56 (11.0)	140 (17.7) **	142 (20.7)	76 (11.3) **
**Physical activity level**						
Low	111 (19.7)	103 (16.9)	95 (20.9)	117 (17.2)	138 (25.7)	92 (14.3)
Medium	266 (47.2)	333 (54.8)	200 (44.0)	369 (54.2)	290 (54.0)	298 (46.2)
High	187 (33.2)	172 (28.3) *	160 (35.2)	195 (28.6) *	109 (20.3)	255 (39.5) **
**BMI categories**						
Normal	263 (44.6)	340 (54.1)	245 (53.0)	322 (43.6)	264 (42.2)	313 (49.7)
Overweight	206 (34.9)	215 (34.2)	156 (33.8)	266 (36.0)	216 (34.6)	238 (37.8)
Obese	121 (20.5)	73 (11.6) **	61 (13.2)	151 (20.4) *	145 (23.2)	79 (12.5) **
**Mediterranean diet adherence**						
Low	362 (57.1)	356 (52.0)	281 (55.3)	415 (52.5)	387 (56.5)	311 (46.3)
Medium	212 (33.4)	252 (36.8)	167 (32.9)	294 (37.2)	260 (38.0)	259 (38.6)
High	60 (9.5)	76 (11.1)	60 (11.8)	82 (10.4)	38 (5.5)	101 (15.1) **

Results are expressed as n (%); * denotes *p* < 0.05 for Chi-squared analysis; ** denotes *p* < 0.001 for Chi-squared analysis.

**Table 2:** Background characteristics of the study sample by consumption of individual alcoholic beverages containing polyphenols.

	Red Wine		White Wine		Beer	
	T1	T3	T1	T3	T1	T3
**Sex**						
Male	253 (37.8)	135 (44.4)	390 (40.5)	29 (32.2)	238 (36.6)	298 (46.9)
Female	416 (62.2)	169 (55.6)	573 (59.5)	61 (67.8)	413 (63.4)	337 (53.1) *
**Age group**						
<30	130 (19.4)	24 (7.9)	181 (18.8)	7 (7.8)	97 (14.9)	134 (21.1)
30–44	196 (29.3)	53 (17.4)	244 (25.3)	23 (25.6)	161 (24.7)	195 (30.7)
44–65	197 (29.4)	131 (43.1)	315 (32.7)	40 (44.4)	214 (32.8)	242 (38.1)
>65	147 (21.9)	96 (31.6) **	224 (23.2)	20 (22.2) **	180 (27.6)	64 (10.1) **
**Educational level**						
Low	280 (41.9)	148 (48.7)	387 (40.2)	41 (45.6)	280 (43.0)	165 (26.0)
Medium	239 (35.7)	80 (26.3)	327 (34.0)	28 (31.1)	222 (34.1)	254 (40.0)
High	150 (22.4)	76 (25.0) **	249 (25.9)	21 (23.3) **	149 (22.9)	216 (34.0) **
**Smoking status**						
Non-smoker	426 (63.7)	168 (55.3)	598 (62.1)	47 (52.2)	394 (60.5)	385 (60.6)
Current smoker	148 (22.1)	66 (21.7)	211 (21.9)	21 (23.3)	138 (21.2)	180 (28.3)
Former smoker	95 (14.2)	70 (23.0) **	154 (16.0)	22 (24.4) **	119 (18.3)	70 (11.0) **
**Physical activity level**						
Low	138 (23.9)	42 (16.3)	194 (23.1)	4 (5.4)	133 (24.4)	92 (15.1)
Medium	296 (51.3)	136 (52.7)	441 (52.6)	46 (62.2)	282 (51.6)	296 (48.7)
High	143 (24.8)	80 (31.0) **	204 (24.3)	24 (32.4) **	131 (24.0)	220 (36.2) **
**BMI categories**						
Normal	311 (50.4)	112 (39.7)	418 (46.4)	42 (47.7)	281 (46.2)	285 (48.1)
Overweight	179 (29.0)	119 (42.2)	302 (33.6)	40 (45.5)	198 (32.6)	233 (39.3)
Obese	127 (20.6)	51 (18.1) **	180 (20.0)	6 (6.8) *	129 (21.2)	75 (12.6) *
**Mediterranean diet adherence**						
Low	418 (62.5)	126 (41.4)	575 (59.7)	40 (44.4)	363 (55.8)	340 (53.5)
Medium	209 (31.2)	145 (47.7)	318 (33.0)	33 (36.7)	238 (36.6)	214 (33.7)
High	42 (6.3)	33 (10.9) **	70 (7.3)	17 (18.9) **	50 (7.7)	81 (12.8) *

Results are expressed as n (%); * denotes *p* < 0.05 for Chi-squared analysis; ** denotes *p* < 0.001 for Chi-squared analysis.

**Table 3:** Total and main classes of dietary polyphenols by consumption of polyphenol-rich beverages.

	Tea		Coffee		Fresh Citrus Juice	
	T1	T3	T1	T3	T1	T3
Total polyphenols (mg/d)	650.5 (833.3)	767.4 (430.1) **	713.4 (899.0)	674.3 (396.6) *	669.2 (492.4)	666.4 (798.8)
Flavonoids (mg/d)	208.7 (189.6)	354.5 (222.4) **	276.6 (241.6)	263.8 (194.5) *	265.9 (188.6)	251.8 (217.7)
Phenolic acids (mg/d)	368.9 (302.9)	396.6 (735.8) *	366.5 (276.2)	391.7 (792.0) *	361.7 (404.3)	374.4 (694.8)
Stilbenes (mg/d)	2.1 (4.0)	1.7 (3.4)	1.03 (1.81)	2.3 (4.2) **	2.6 (4.4)	0.9 (1.5) **
Lignans (mg/d)	2.7 (2.8)	2.8 (2.5)	2.9 (3.1)	2.7 (2.3)	2.6 (2.2)	2.8 (3.0)

Results are expressed as mean (SD); * denotes *p* < 0.05 for ANOVA or ANCOVA analysis; ** denotes *p* < 0.001 for ANOVA or ANCOVA analysis.

**Table 4:** Total and main classes of dietary polyphenols by consumption of alcoholic beverages containing polyphenols.

	Red Wine		White Wine		Beer	
	T1	T3	T1	T3	T1	T3
Total polyphenols (mg/d)	535.2 (357.1)	896.6 (589.6) **	584.2 (458.9)	964.1 (524.2) **	606.1 (529.4)	681.1 (455.4) *
Flavonoids (mg/d)	206.0 (171.9)	381.6 (182.9) **	229.5 (177.7)	444.9 (210.1) **	250.7 (197.1)	265.3 (208.1)
Phenolic acids (mg/d)	293.0 (261.3)	465.1 (548.3) **	317.1 (372.6)	464.0 (434.4) **	318.0 (437.6)	370.8 (306.6) *
Stilbenes (mg/d)	0.1 (0.4)	8.4 (4.8) **	1.5 (3.6)	7.7 (4.7) **	1.8 (4.0)	1.7 (2.3)
Lignans (mg/d)	2.4 (1.9)	3.2 (2.6) **	2.6 (2.4)	3.6 (2.9) *	2.6 (2.2)	2.7 (2.9)

Results are expressed as mean (SD); * denotes *p* < 0.05 for ANOVA or ANCOVA analysis; ** denotes *p* < 0.001 for ANOVA or ANCOVA analysis.

**Table 5:** Association between polyphenol-rich beverages consumption and metabolic outcomes in the study sample.

	Hypertension			Type-2 Diabetes			Dyslipidemia		
	T1	T2	T3	T1	T2	T3	T1	T2	T3
**Tea**									
Model 1 ^a^	1	1.45 (1.14–1.84)	0.86 (0.69–1.07)	1	1.42 (0.89–2.27)	1.03 (0.66–1.63)	1	0.94 (0.67–1.30)	0.75 (0.50–1.12)
Model 2 ^b^	1	1.42 (1.06–1.90)	1.00 (0.76–1.30)	1	1.19 (0.69–2.05)	1.44 (0.84–2.48)	1	0.88 (0.59–1.31)	0.84 (0.64–1.10)
Model 3 ^c^	1	1.39 (1.04–1.86)	0.95 (0.73–1.25)	1	1.15 (0.67–1.98)	1.39 (0.81–2.40)	1	0.90 (0.60–1.34)	0.91 (0.64–1.28)
**Coffee**									
Model 1 ^a^	1	1.25 (0.97–1.60)	0.96 (0.76–1.22)	1	0.99 (0.60–1.64)	0.92 (0.57–1.47)	1	1.08 (0.77–1.50)	0.90 (0.66–1.22)
Model 2 ^b^	1	1.01 (0.76–1.36)	0.66 (0.50–0.88)	1	0.72 (0.40–1.31)	0.74 (0.42–1.30)	1	0.80 (0.53–1.19)	0.64 (0.43–0.95)
Model 3 ^c^	1	1.00 (0.74–1.34)	0.64 (0.48–0.86)	1	0.72 (0.39–1.30)	0.74 (0.42–1.31)	1	0.79 (0.53–1.17)	0.64 (0.43–0.95)
**Fresh citrus juice**									
Model 1 ^a^	1	0.63 (0.45–0.86)	0.65 (0.52–0.81)	1	1.44 (0.85–2.43)	0.31 (0.17–0.56)	1	0.67 (0.45–1.02)	0.29 (0.21–0.41)
Model 2 ^b^	1	0.73 (0.51–1.06)	0.79 (0.61–1.02)	1	1.07 (0.67–1.73)	0.41 (0.22–0.77)	1	0.83 (0.52–1.32)	0.33 (0.22–0.49)
Model 3 ^c^	1	0.71 (0.49–1.03)	0.80 (0.62–1.04)	1	1.17 (0.62–2.22)	0.43 (0.23–0.80)	1	0.84 (0.53–1.35)	0.32 (0.22–0.48)

Results are expressed as OR (95% CI); ^a^ Adjusted for total energy intake and all beverages investigated; ^b^ Adjusted as model 1 plus age, sex, educational status, smoking status, physical activity level; ^c^ Adjusted as model 2 plus adherence to the Mediterranean diet.

**Table 6:** Association between intake of alcoholic beverages containing polyphenols and metabolic outcomes in the study sample.

	Hypertension			Type-2 Diabetes			Dyslipidemia		
	T1	T2	T3	T1	T2	T3	T1	T2	T3
**Red wine**									
Model 1 ^a^	1	0.61 (0.44–0.85)	0.71 (0.52–0.97)	1	0.43 (0.25–0.73)	0.65 (0.39–1.09)	1	0.62 (0.43–0.90)	0.81 (0.56–1.16)
Model 2 ^b^	1	0.55 (0.37–0.82)	0.61 (0.41–0.90)	1	0.34 (0.18–0.64)	0.63 (0.35–1.15)	1	0.41 (0.26–0.66)	0.74 (0.47–1.16)
Model 3 ^c^	1	0.57 (0.38–0.86)	0.61 (0.41–0.90)	1	0.37 (0.19–0.71)	0.65 (0.36–1.18)	1	0.39 (0.24–0.63)	0.71 (0.45–1.12)
**White wine**									
Model 1 ^a^	1	0.78 (0.61–1.00)	0.67 (0.41–1.10)	1	0.89 (0.56–1.42)	0.42 (0.15–1.17)	1	0.82 (0.60–1.13)	0.85 (0.47–1.53)
Model 2 ^b^	1	0.85 (0.63–1.14)	0.69 (0.38–1.25)	1	0.76 (0.44–1.30)	0.15 (0.03–0.76)	1	0.79 (0.54–1.16)	1.03 (0.51–2.09)
Model 3 ^c^	1	0.85 (0.63–1.15)	0.69 (0.38–1.26)	1	0.75 (0.43–1.28)	0.14 (0.03–0.75)	1	0.81 (0.55–1.18)	1.00 (0.49–2.04)
**Beer**									
Model 1 ^a^	1	0.79 (0.62–1.00)	0.59 (0.46–0.76)	1	0.88 (0.58–1.34)	0.38 (0.22–0.66)	1	0.94 (0.71–1.26)	0.78 (0.56–1.08)
Model 2 ^b^	1	0.78 (0.58–1.04)	0.61 (0.45–0.83)	1	0.94 (0.57–1.56)	0.51 (0.28–0.91)	1	1.03 (0.71–1.48)	1.13 (0.77–1.67)
Model 3 ^c^	1	0.78 (0.58–1.04)	0.61 (0.45–0.83)	1	0.94 (0.57–1.56)	0.51 (0.28–0.92)	1	1.05 (0.73–1.52)	1.14 (0.77–1.68)

Results are expressed as OR (95% CI); ^a^ Adjusted for total energy intake and all beverages investigated; ^b^ Adjusted as model 1 plus age, sex, educational status, smoking status, physical activity level; ^c^ Adjusted as model 2 plus adherence to the Mediterranean diet.

**Table 7:** Dietary factor distribution through sample-level grouping by polyphenol-rich beverage consumption.

	Polyphenol-Rich Beverage Consumption			
	T1	T2	T3	*p_trend_*
Tea (mL/d)	77.5 (180.3)	75.7 (135.3)	68.0 (136.9)	0.294
Coffee (mL/d)	47.6 (51.9)	58.9 (44.9)	67.6 (39.5)	<0.001
Red wine (mL/d)	6.0 (26.7)	43.1 (91.3)	53.9 (80.4)	<0.001
White wine (mL/d)	3.4 (37.0)	8.7 (34.8)	26.4 (44.1)	<0.001
Beer (mL/d)	3.3 (14.3)	37.1 (104.7)	100.1 (160.0)	<0.001
Fresh citrus juice (mL/d)	11.8 (49.3)	25.2 (70.1)	41.0 (92.9)	<0.001
Energy intake (kcal/d)	2051.0 (717.3)	2039.8 (798.6)	2246.8 (905.6)	<0.001
Energy intake (kJ/d)	8296.3 (2938.4)	8238.0 (3324.9)	9163.8 (3741.4)	<0.001
**Macronutrients**				
Carbohydrates (g/d)	309.0 (121.8)	298.2 (129.9)	317.1 (129.9)	0.209
Fiber (g/d)	31.4 (11.9)	31.1 (13.3)	32.2 (16.5)	0.319
Protein (g/d)	85.2 (28.4)	82.1 (27.5)	86.4 (31.5)	0.361
Fat (g/d)	60.2 (22.0)	59.9 (26.5)	67.6 (32.9)	<0.001
Cholesterol (mg/d)	195.6 (100.7)	182.4 (82.3)	197.9 (88.3)	0.472
SFA %	23.5 (9.3)	22.4 (9.4)	25.2 (10.4)	0.002
MUFA %	25.2 (8.0)	24.6 (8.5)	26.3 (9.9)	0.030
PUFA %	11.1 (4.7)	12.1 (6.5)	14.9 (9.7)	<0.001
Total Omega-3 g	1.7 (0.8)	1.6 (0.9)	1.6 (0.7)	0.019
**Micronutrients**				
Vitamin A (Retinol)	831.2 (357.8)	891.6 (438.3)	856.5 (455.4)	0.473
Vitamin C (mg/d)	140.9 (80.7)	159.0 (84.9)	167.4 (120.6)	<0.001
Vitamin E (mg/d)	8.2 (2.9)	8.5 (3.1)	9.0 (3.8)	<0.001
Vitamin B12	6.2 (4.5)	6.1 (3.8)	6.6 (6.1)	0.093
Vitamin D	5.6 (6.1)	5.3 (4.7)	5.2 (4.7)	0.159
Sodium (mg/d)	2770.1 (1183.4)	2752.6 (1008.2)	3036.7 (1137.3)	<0.001
Potassium (mg/d)	3471.2 (1109.6)	3627.4 (1276.0)	3805.9 (1644.6)	<0.001
**Polyphenols**				
Total polyphenols	516.9 (339.5)	698.4 (775.0)	715.4 (475.8)	<0.001
Flavonoids	211.3 (189.3)	263.4 (186.4)	286.3 (217.6)	<0.001
Phenolic acids	271.7 (242.4)	393.1 (701.3)	381.6 (316.0)	0.002
Stilbenes	0.4 (1.2)	2.1 (4.0)	2.7 (3.7)	<0.001
Lignans	2.4 (1.8)	2.9 (2.7)	2.7 (2.8)	0.076

Results are expressed as mean (SD).

**Table 8:** Association between sample-level grouping by polyphenol-rich beverage consumption and metabolic outcomes.

	Hypertension			Type-2 Diabetes			Dyslipidemia		
	T1	T2	T3	T1	T2	T3	T1	T2	T3
**Polyphenol-rich beverage** **consumption**									
Model 1 ^a^	1	0.73 (0.57–0.92)	0.57 (0.44–0.73)	1	0.54 (0.36–0.81)	0.41 (0.26–0.66)	1	0.67 (0.51–0.89)	0.41 (0.29–0.57)
Model 2 ^b^	1	0.80 (0.60–1.08)	0.70 (0.51–0.95)	1	0.75 (0.45–1.26)	0.71 (0.40–1.26)	1	0.82 (0.57–1.17)	0.73 (0.49–1.09)
Model 3 ^c^	1	0.79 (0.59–1.06)	0.69 (0.50–0.94)	1	0.71 (0.42–1.19)	0.66 (0.37–1.18)	1	0.82 (0.57–1.18)	0.73 (0.49–1.10)

Results are expressed as OR (95% CI); ^a^ Adjusted for total energy intake and all beverages investigated; ^b^ Adjusted as model 1 plus age, sex, educational status, smoking status, physical activity level; ^c^ Adjusted as model 2 plus adherence to the Mediterranean diet.

## Supplementary Materials

The following are available online at https://www.mdpi.com/2304-8158/10/2/383/s1, Table S1: Dietary intake of polyphenol-rich beverages by tertile of consumption.
