# Supplementary material for: Polyphenol-Rich and Alcoholic Beverages and Metabolic Status in Adults Living in Sicily, Southern Italy
Source: Foods. 2021 Feb 9;10(2):383. doi: 10.3390/foods10020383 (PMC7916404; doi:10.3390/foods10020383)
Supplement: Supplementary file 1 [file foods-10-00383-s001.zip › foods-1080744-supplementary.pdf]

foods

Supplementary Table 1. Dietary intake of polyphenol-rich beverages by tertile of consumption.

	**Individual polyphenol-rich beverage consumption**		
	T1	T2	T3
		Mean (SD)	
Tea (ml/d)	0.0 (0.0)	17.2 (8.7)	179.7 (197.3)
Coffee (ml/d)	8.5 (8.6)	36.3 (6.1)	103.6 (29.4)
Red wine (ml/d)	0.0 (0.0)	16.9 (17.4)	180.7 (110.9)
White wine (ml/d)	11.9 (37.1)	14.4 (44.8)	15.0 (34.2)
Beer (ml/d)	0.0 (0.0)	14.7 (5.9)	131.1 (179.1)
Fresh citrus juice (ml/d)	0.0 (0.0)	6.6 (0.0)	64.5 (107.5)
